# Agreement Document for the Study of Leishmaniasis from a One Health Approach in Spain

**DOI:** 10.3390/tropicalmed10090269

**Published:** 2025-09-18

**Authors:** Joaquina Martín-Sánchez, Jose M. Requena, Montserrat Gállego, Maribel Jiménez, Ricardo Molina, Paul Nguewa, Francisco Morillas-Márquez, José M. Pérez-Victoria, Begoña Monge-Maillo, Manuel Morales-Yuste, Clotilde Marín

**Affiliations:** 1 Departamento de Parasitología, Universidad de Granada, 18071 Granada, Spain; joaquina@ugr.es (J.M.-S.); fmorilla@ugr.es (F.M.-M.); 2 Centro de Biología Molecular Severo Ochoa (CSIC-UAM), Departamento de Biología Molecular, Universidad Autónoma de Madrid, 28049 Madrid, Spain; jmrequena@cbm.csic.es; 3 Secció de Parasitologia, Departament de Biologia, Sanitat i Medi Ambient, Facultat de Farmàcia i Ciències de l’Alimentació, Universitat de Barcelona, 08028 Barcelona, Spain; mgallego@ub.edu; 4 Instituto de Salud Global de Barcelona (ISGlobal), 08036 Barcelona, Spain; 5 Centro de Investigación Biomédica en Red Enfermedades Infecciosas (CIBERINF), Instituto de Salud Carlos III, 28029 Madrid, Spain; mjimenez@isciii.es (M.J.); rmolina@isciii.es (R.M.); 6 Laboratorio de Entomología Médica, Centro Nacional de Microbiología, Instituto de Salud Carlos III, Majadahonda, 28029 Madrid, Spain; 7 Departamento de Microbiología y Parasitología, Instituto de Investigación Sanitaria de Navarra (IdisNA), Universidad de Navarra, 31008 Pamplona, Spain; panguewa@unav.es; 8 Instituto de Parasitología y Biomedicina ‘López-Neyra’, (IPBLN-CSIC), Health Technology Park (PTS Granada), 18016 Granada, Spain; josepv@ipb.csic.es; 9 Centro de Referencia Nacional para Enfermedades Tropicales, Servicio de Enfermedades Infecciosas, Hospital Universitario Ramón y Cajal, IRYCIS, CIBER de Enfermedades Infecciosas (CIBERINFEC), Instituto de Salud Carlos III, 28029 Madrid, Spain; begomongem@gmail.com

**Keywords:** *Leishmania*, *Leishmania infantum*, leishmaniasis, One Health

## Abstract

**Background**: Leishmaniasis, a vector-borne disease caused by the parasite *Leishmania*, is a public health concern in Europe. Although Spain is an endemic country, it lacks a national surveillance network. To address this, the creation of a research and surveillance network is proposed, which would integrate data from various fields and enhance control and public health strategies. **Objectives**: The first objective is to gather epidemiological data on vectors, reservoirs, and transmission rates in Spain, with a particular focus on the role of dogs and wildlife as primary reservoirs. The plan includes establishing forums, databases, and specialised training programmes. The second objective is to improve case surveillance and reporting by establishing a comprehensive national registry. Implementing molecular diagnostics and bioinformatics tools will aid analysis of retrospective data and tracking of disease evolution. The third objective is to develop a standardised method for the molecular characterisation of *Leishmania*, using techniques such as PCR and genomic sequencing to detect virulence and resistance patterns. Key actions include collecting isolates, sharing analytical tools and providing genomic training. The fourth objective is to identify new therapeutic targets in response to toxicity and resistance to current treatments by studying specific enzymes (FeSOD, HDAC), metabolic pathways, and natural products. Ultimately, establishing the Leishmaniasis Surveillance Network will support a “One Health” approach and promote interdisciplinary collaboration towards a national control plan. **Methods**: We created the Working Group on Leishmaniasis funded by the Spanish Society of Tropical Medicine and International Health as a leishmaniasis forum for meetings and discussions on weaknesses and gaps we find in the knowledge and management of the infection based on the background of each academic and healthcare research group. **Expected Impact**: This study will promote the articulation of a Leishmaniasis Surveillance Network aiming to bring together the main stakeholders in the research and management of leishmaniasis in Spain.

## 1. Introduction

Infections caused by protozoa of the genus *Leishmania* have a particular impact on human health because they cause leishmaniasis that is emerging or re-emerging in the European Union and neighbouring countries. Leishmaniasis is a vector-borne, poverty-associated disease, whose vector is the female phlebotomine sand fly, a small dipteran insect, about 3 mm in size, with a gibbous body and lanceolate wings, which remain in a “V” shape when resting. After transmission, these parasites can promote in the host a wide clinical spectrum, ranging from no symptoms or localised skin ulcers to a potentially fatal systemic disease, known as visceral leishmaniasis. Leishmaniasis is endemic in 99 countries with 350 million people at risk and an estimated global mortality of 59,000 deaths annually [1]. The burden of disease is estimated at 2,356,000 disability-adjusted life years (DALYs), a significant figure in the context of infectious diseases. Annual reported cases are estimated at 50,000–90,000 visceral cases and 600,000–1,000,000 new cutaneous/mucosal cases, but the true incidence is difficult to estimate [1,2]. According to WHO (World Health Organization), leishmaniasis remains a major public health problem in four eco-epidemiological regions: the Americas, East Africa, North Africa, and West and South-East Asia [3]. However, in Spain and other European Mediterranean countries, zoonotic leishmaniasis due to *Leishmania* (L.) *infantum* has been a known endemism for decades. This species causes visceral (VL), cutaneous (CL), and mucosal (ML) leishmaniasis in humans, as well as canine leishmaniasis (CanL), with dogs being the main domestic reservoir [4,5,6,7]. Several environmental factors, such as climate change, increased urbanisation, or colonisation of urban areas by wildlife, all of which are part of global change, are causing significant changes in the epidemiology of leishmaniasis because of variations in the availability of its vectors and reservoirs [4,8,9,10,11,12]. Thus, rabbits and hares have been shown to play a key role in the transmission of the parasite and to impact human leishmaniasis incidence [13,14,15,16,17].

An integrated “One Health” approach to tackle this zoonosis, incorporating the latest advances in public, veterinary, and environmental health, would undoubtedly offer many advantages and new options for disease control. In addition, many cases of human leishmaniasis, especially cutaneous and mucosal forms, remain undiagnosed or undeclared in our country [18,19,20]. This lack of a national registration system is reflected in the latest WHO report on leishmaniasis endemicity, where Spain appears as “no data” [3]. On the other hand, regarding CanL, significant progress has been made to increase the efficiency of its management and minimise its impact on public health [21]. However, it is necessary to include in control strategies measures targeting vertebrate hosts other than canids.

**Figure 1:**
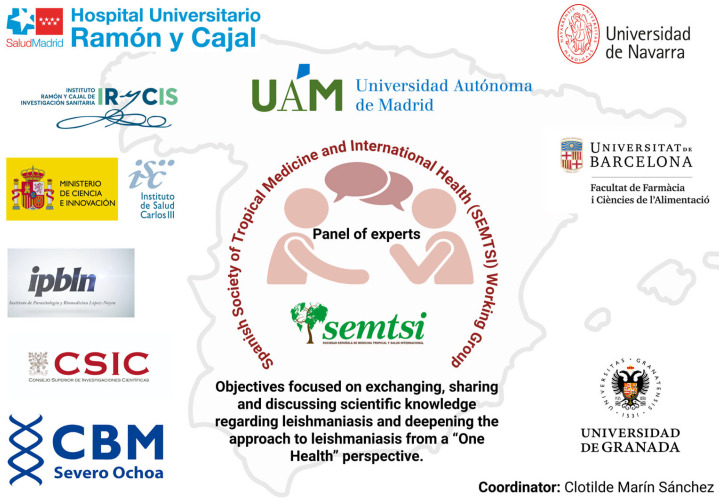
Participating institutions in the Leishmaniasis Working Group. Created in BioRender (https://BioRender.com/kmtkg27, accessed on 8 July 2025).

In 2021, the Working Group on Leishmaniasis was created, funded by the Spanish Society of Tropical Medicine and International Health (SEMTSI, https://www.semtsi.es/grupos-de-trabajo/, accessed on 8 July 2025) and composed of about twenty professionals from different fields of research, health, and management (Figure 1). The development of the mentioned group has been a forum for discussion in the seven meetings held. Our main concern has highlighted the absence of a comprehensive national surveillance programme, which has prompted us to produce this agreement or consensus document to demonstrate the urgent need for our country to have a research network based on a comprehensive approach that contributes new knowledge and strengthens the fight against this parasitic disease.

The **general objective** of this working group is to facilitate the transfer of basic research results generated by Spanish groups specialised in different areas of research on leishmaniasis to clinical and veterinary applications that lead to the integrated control of this zoonosis in our country. Spain is an endemic country for leishmaniasis, with a significant number of human cases and with CanL as a first-level veterinary problem. However, there is neither a national surveillance network nor structure aimed at reducing the negative impact of leishmaniasis on public and veterinary health. This is the first time an attempt has been made to integrate the main leishmaniasis research groups in our country into a network, to promote the association of their research results and to promote coordinated measures to control this parasitosis.

To this end, a series of specific objectives are set out, based on the background of the Spanish research group and the milestones achieved to date.

## 2. Objective 1: To Efficiently Integrate the Basic Knowledge Derived from Epidemiologic Studies on Leishmaniasis Carried out in Areas of Different Endemicities in Spain

The epidemiologic data generated by the groups will be analysed and reconciled to deepening our understanding of vector/reservoir/parasite interactions and their relationship with environmental changes under a “One Health” view.

As leishmaniasis is a vector-borne disease, the presence of phlebotomine sand flies is considered the main risk for the occurrence of cases in each area. Non-vector transmission routes such as blood transfusion, needle reuse, and sexual and vertical transmission have been poorly described in endemic areas, but some studies show evidence that these alternative routes may have a greater impact on transmission than assumed [22,23,24]. Recent studies suggest that vertical transmission appears to play an important role in the spread of leishmaniasis in prolific animal hosts [25].

Leishmaniasis is caused by *L. infantum* in southwestern Europe, where the incidence in humans is low despite a high prevalence in dogs, the main domestic reservoir. Studies of risk factors for human infection with *L. infantum* have yielded conflicting results, although it is still widely believed that owners of infected dogs and other members of the household environment, in which cases of the disease occur, may be at high risk of infection.

*L. infantum* infection in humans results in clinical disease in only a fraction of the total number of infected individuals [26,27,28,29], which has also been demonstrated in the canine population [30,31,32]. While the epidemiologic role of asymptomatic infected individuals is not fully understood [33], asymptomatic dogs have been shown to be infective for phlebotomine sand fly vectors [34,35,36], although there are conflicting results [37]. A growing number of studies indicate that leishmaniasis affects many mammalian species in Europe, both domestic and wild [13,14,15,16,17,38,39,40,41], and interactions may occur. The understanding of the role of wildlife in the epidemiology of leishmaniasis will be helpful to design interventions to reduce the prevalence of this parasitosis. Human interventions have been shown to alter the dynamics of *Leishmania* transmission, potentially increasing exposure to infected vectors and susceptible reservoirs, or facilitating interactions between domestic and wild transmission cycles. Various factors related to urbanisation and changes in land use have led to an increase in the populations of leporids and *Phlebotomus* (P.) *perniciosus*, the main vector of the parasite in Spain, in municipalities in the south-west of the Community of Madrid. This phenomenon has favoured the transmission of the parasite among Iberian hares and, to a lesser extent, wild rabbits in the green areas surrounding the affected municipalities [13,15], leading to the largest outbreak of human leishmaniasis in Europe, with 850 cases reported from 2010 to date [42]. In another classical endemic area, the Province of Granada, a positive correlation was found between the incidence of human leishmaniasis and the parasite load in the ear skin of wild rabbits. The same authors have shown that the genotypes of *L. infantum* present in these rabbits are the same as those identified in humans, supporting the theory of wild rabbits as relevant reservoirs of this parasite [17].

Effective control of the transmission of this phlebotomine sand fly vector-borne zoonosis requires integrated approaches that focus on all the agents involved in transmission: (i) the parasite, (ii) the network of hosts that cooperate in its maintenance, with the dog as the main domestic reservoir and lagomorphs as the main wild reservoir, and (iii) the phlebotomine sand fly vector, which is strongly conditioned by environmental characteristics, highlighting the importance of ecological relationships.

Three species of phlebotomine sand flies of the subgenus *Larroussius* are involved in the transmission of *L. infantum* in the Iberian Peninsula: *P. perniciosus* (the main vector), *P. ariasi*, and *P. langeroni*; the parasite has been isolated or *Leishmania* DNA has been detected in these species [8,9,11,43,44,45,46,47,48,49,50,51,52,53,54]. Although PCR (polymerase chain reaction) is a useful technique for selecting vector species in each area, especially when analysing pools of samples [55], the incrimination of a vector species based solely on the detection of *Leishmania* DNA should be treated with caution. Such DNA could result from the casual ingestion of blood from a parasitized animal without the development of the biological cycle in the sand fly [56]. The presence of two sympatric vector species in the same focus is noteworthy [45,47]. Changes in the distribution of phlebotomine sand fly are essential for determining the likelihood of expansion/redistribution of leishmaniasis risk areas [10,57]. Moreover, at the local level, they may condition the uneven distribution of human leishmaniasis cases in endemic areas. All this supports the need for updated data on vector species at the peridomestic level, both intra and peridomestic, to better understand the transmission dynamics of *L. infantum*. Definitively, the transmission of leishmaniasis is linked to specific ecosystems. Although the links between environmental factors and changes in the distribution of *L. infantum* vectors in Spain have been extensively studied [8,9,10,11,12], the drivers for an increased transmission are not always understood, even though they are related to the presence of the vector and highly infected hosts [16]. Therefore, an in-depth study of other vertebrate hosts, such as cats, rodents, horses, goats, and wild carnivores in general, will contribute to a better understanding of the epidemiology of the disease.

### Proposed Actions to Achieve Objective 1

- Organisation of discussion forums.
- Creation of a global database and application/validation of statistical models and GIS (geographical information systems) to predict the risk of leishmaniasis in our country, including vectors, dogs, and other hosts.
- To promote entomological and animal case surveillance.
- To include in the database results from other groups that have conducted epidemiological studies on leishmaniasis in our country.
- Training of network participants in vector/parasite/dog surveillance methodology and recognition of other animal hosts.

## 3. Objective 2: Consolidation of a Stable Reporting Structure for Human Leishmaniasis Cases and Promotion of the Incorporation of Optimised Detection Protocols

Epidemiological surveillance of leishmaniasis in Spain began in 1982, when the disease was included in the list of notifiable diseases. In 1995, the National Epidemiological Surveillance Network (RENAVE) was created through Royal Decree 2210/1995 (BOE No. 21 of 24 January 1996), classifying leishmaniasis as a regionally endemic disease and excluding its notification in the autonomous communities that are considered non-endemic. It was no longer mandatory to report leishmaniasis in regions considered non-endemic. In 2015, with the Order SSI/445/2015 (BOE No. 65 of 17 March 2015), leishmaniasis became notifiable again throughout the country.

Since 2016, the evolution of the number of autochthonous cases has been relatively stable, with an incidence rate (IR) between 0.76 and 0.9 cases per 100,000 inhabitants. The highest IRs were most frequently recorded in the Balearic Islands, Valencia, Madrid, and Murcia [58]. However, there is significant underreporting of cases—close to 50%—affecting all clinical forms but particularly ML and CL [5,18,19,20]. A recent study estimated the national incidence of human leishmaniasis using data collected in the RENAVE and the Minimum Basic Data Set with hospital care episodes (CMBD). This work estimated underreporting in Spain to be 18% for VL, 53.9% for cases of CL, and 70% for mucocutaneous leishmaniasis (MCL) [59].

Apart from autochthonous cases by *L. infantum*, an increase in the presence of imported leishmaniasis has been detected in Spain as in other European countries [60,61,62,63,64]. These cases come mainly from Latin American and Maghreb countries and several *Leishmania* species area incriminated. Some of these cases are recorded in official epidemiological bulletins, but no data are included on the origin of the patients nor the species incriminated.

A national registry of leishmaniasis cases, collected prospectively, dynamically, and with detailed clinical data, would help in the planning of control measures that can be applied in real time in healthcare services, and would provide a detailed picture of the clinical, diagnostic, therapeutic, and prognostic characteristics of leishmaniasis in our environment. This is an important learning experience for the improvement of clinical case management and the development of leishmaniasis research. Thus, in March 2021, the National Reference Centre for Tropical Diseases (CSUR-Trop) was designated as a World Health Organization Collaborating Centre (WHOCC) for the clinical management of leishmaniasis “SPA-55” (https://leishinfowho-cc55.es/, accessed on 16 September 2025) under the direction of Dr. Begoña Monge-Maillo and Dr. Rogelio López-Vélez. One of the objectives of the WHOCC is to provide technical support to the WHO to improve surveillance of leishmaniasis at individual and sub-national levels. To this end, the National Leishmaniasis Network (ReNLeish) was created by CSUR-Trop in January 2022 (contact: leish.spain@gmail.com). ReNLeish was created with the aim of collecting cases of leishmaniasis but is also presented as a platform for sharing complex cases, contributing to the formation of health professionals and the general population, and promoting collaborative research in leishmaniasis. For this purpose, the information stored in the central database is obtained from medical records or databases used in clinical practice for patient care and follow-up (within the research data management tool known as *Research Electronic Data Capture* or RedCAP). There is no time limit on the affiliation of new collaborating centres to the network. The affiliation must be approved by the Research Ethics Committees of each collaborating centre. These entities include hospitals, specialised centres, and primary care centres. The data provided allow an analysis to be performed that shows the situation of leishmaniasis in Spain through publishable results. By December 2024, 43 centres from 17 different provinces in Spain had been included, 27 of which had registered a total of 335 cases of leishmaniasis. However, the under-reporting previously mentioned in this document confirms that there is still a need to improve leishmaniasis case registration in Spain with comprehensive and real-time data. Therefore, although this new network represents a major step forward, we propose to complement its creation with several measures.

### Proposed Actions to Achieve Objective 2

- To optimise the design of the information technology (IT) tool for efficient and simplified case collection and incorporation of new data of epidemiological interest.
- The possibility of providing retrospective cases in addition to prospective cases.
- To include not only clinical but also other epidemiological groups in the database.
- The possibility of monitoring the temporal evolution of the incidence and generating distribution maps.

## 4. Objective 3: Development of a Reference Method for Rapid and Complete Molecular Characterisation of Leishmania Isolates

The availability of such a method will allow the completion of an extensive database of parasite isolates obtained from circulating samples in Spain. In this way, it would be possible to detect molecular patterns associated with different degrees of virulence, tropism for different reservoirs, and drug resistance.

The differentiation of *Leishmania* species is of interest from several perspectives, particularly in the fields of healthcare, taxonomy, and epidemiology. As there is no morphological characteristic that allows species to be distinguished within the genus, a variety of techniques based on biochemical, genetic or proteomic methodologies have been developed [65,66].

Currently, there is no consensus on their selection, and each laboratory uses those that are best suited to its purposes. Nevertheless, to maintain the conciliatory line supported by the One Health approach, it would be advisable to develop an integrated genotyping procedure that could be used by all researchers and health professionals, including veterinarians and public health professionals. Isoenzyme analysis, also known as multilocus enzyme electrophoresis (MLEE) [67], was a pioneering technique in the characterisation of *Leishmania*, but this technique is now being replaced by methods with greater discriminatory power. One of the primary drawbacks of isoenzyme typing is its laborious nature and the fact that it is a homemade technique. This technique requires the mass cultivation of the strains under study, along with the use of reference strains for comparisons. Nevertheless, isoenzyme electrophoresis has been extensively used in Spain and has provided valuable contributions, such as demonstrating that *L. infantum* is the only endemic species in our country and is responsible for both cutaneous and visceral clinical manifestations [48,68,69,70,71,72], and as we now know, also for ML and CML cases [73]. This technique also revealed the existence of a significant intraspecific isoenzyme variability (zymodemes), being greater in VIH coinfected patients, whilst in dogs a viscerotropic zymodeme predominates [48,68,69,70,71,74,75]. The presence of two zymodemes in the same patient and in dog has been found [76,77]. Few studies have been conducted with strains isolated from sand flies that also showed this variability [45,48,69,71]. The characterisation of these zymodemes has allowed to suggest the transmission of the parasite through syringes shared by injecting drug users [74], a fact subsequently demonstrated experimentally [23], and to suggest the existence of sexual reproduction of the parasite within the phlebotomine sand fly vector [71,78].

The genotyping methods of this parasitic protozoan, which are now more widely used, are based on the study of the gene sequence, both of a single locus and of multiple loci, by sequencing or size analysis of a polymorphic regions in genes such as *hsp70*, *ITS*, *RNA7SL*, *kDNA*, *gp63*, and *cytb*, among others [63]. While these genes are useful for typing different species of the genus Leishmania, they lack the discriminatory power to address local or regional epidemiological questions related to *L. infantum* strain variability.

The molecular characterisation of *L. infantum* isolates by analysis of genomic molecular markers (e.g.*, hsp70*, *mpi* and *ITS1*) by PCR and nucleotide sequencing, or by markers such as Spliced leader, *pgd*, *7SL* RNA, *gp63*, and *cytb*, were not able to detect polymorphisms within *L. infantum* species [72,79]. In contrast, markers that do discriminate *L. infantum* strains are the *K26* gene (based on amplicon size) and *kDNA* (PCR sequencing) [17,80,81].

The random amplification of polymorphic DNA (RAPD) technique facilitates the differentiation of species and the demonstration of intraspecific variability within *L. infantum*. However, it is a laborious technique, and the results are influenced by experimental conditions [82]. Also, the analysis of strains by microsatellite analysis showed a great variability and heterogeneity of strains from vectors and vertebrates in the same focus but, as for RAPD, no correlation exists between molecular and enzymatic techniques [83]. Proteomic techniques, which are based on the study of whole proteome, have more recently been incorporated as genotyping methods for *Leishmania*. Specifically, matrix-assisted laser desorption/ionisation mass spectrometry (MALDI-TOF) is used for this purpose. This approach offers several key advantages, including its rapid results output. However, it should be noted that the process necessitates the execution of cultures and can only facilitate identification at the complex level [66].

Advancements in nucleic acid sequencing techniques, coupled with their reduced cost, have made it possible to utilise large genomic regions (and even entire genomes) in the typing of strains shared by vectors, reservoirs, and patients. Genome assemblies are now available for 18 different *Leishmania* species (TriTrypDB.org), including the reference genome of *L. infantum*, generated from the autochthonous strain JPCM5 [84].

Recent studies have demonstrated the discriminatory power of intraspecific typing based on genomic sequences. For instance, the study of the mitochondrial genome of a collection of *L. infantum* strains has enabled the identification of several lineages distributed throughout the Mediterranean basin. Furthermore, it has been established that the strains circulating in Brazil are related to the reference JPCM5 strain [85]. In line with this conclusion, Teixeira et al. (2017) analysed the genomic sequences of 20 *L. infantum* isolates collected in Brazil, concluding that there is high homogeneity among them (99.95% sequence identity) and that they have evolutionary proximity to the reference JPCM5 strain [86].

The value of sequence data analysis at the genomic level has been demonstrated in recent publications. For instance, a study of *L. donovani* isolates in Ethiopia revealed that VL episodes frequently experienced by individuals co-infected with human immunodeficiency virus (HIV) are not the result of new reinfections but a consequence of reactivation of previous infections [87]. The findings of this study corroborate the assumption that treatment of VL does not result in a sterilising cure, but the parasite remains in a dormant state that is reactivated when the immune system is compromised.

As Domagalska and Dujardin (2020) have indicated, while whole genome sequencing is of great value in the molecular surveillance of leishmaniasis. It is important to design methodologies that allow the parasite genome sequence to be obtained directly from host tissue samples, rather than using cultured isolates [88]. This approach is useful because axenic culture conditions may favour chromosomal amplifications/deletions. Aneuploidy mosaicism has been observed in cell cultures of *Leishmania* promastigotes, a consequence of the high plasticity of the Leishmania genome and the less stringent conditions the parasite encounters in axenic media compared to the mammalian host environment [89].

On the other hand, the use of large-scale sequencing methods (both genomic DNA and transcriptome) has great potential to rapidly identify biomarkers and resistance determinants. These approaches are now being used for drug resistance studies. Thus, processes such as modulation of gene expression, changes in gene dosage, variations in chromosome number or selection of sequence polymorphisms, and the phenotype of parasite resistance to drugs used to treat leishmaniasis have been demonstrated and experimentally correlated [90,91].

In short, since not all characterisation techniques provide the same degree of discrimination, the choice of the most appropriate methodology can be made according to the specific purpose of characterisation (clinical and epidemiological management of the disease or epidemiological field studies as an example) [63,79]. In healthcare settings, the characterisation techniques must allow adequate prognosis to guide a correct therapeutic management of the disease. At least species-level differentiation is required, as there are inter-species differences in clinical assessment, likelihood of complications, and response to treatment. It is relevant that the diagnostic techniques can discriminate *Leishmania* species in endemic areas where different species coexist, and in places where imported cases occur due to population migration [63]. In this regard, molecular techniques such as species-specific PCR, PCR-RFLP (more usually ITS-1) or PCR-amplicon sequencing offer the possibility of direct identification from the clinical sample without the need for culture, simultaneously serving diagnosis and species identification. Many different PCR targets have been described, but more comparative studies are still needed to improve and optimise molecular diagnostics, considering that the diversity of epidemiological scenarios may affect their efficiencies [92,93]. On the contrary, to further study the transmission dynamics of *Leishmania* and to identify the source of infection, a high discriminatory capability of parasites—beyond species assignment—is required, so it may be very interesting to expand the use of large-scale sequencing technologies.

Finally, it is important to know whether potential cases of treatment failure are due to the emergence of parasites resistant to the drugs used. Increasing drug resistance affects treatment outcomes, and understanding their causes, spread, and impact will help us manage the risks involved. Having clinical isolates—before and after treatment—is an important challenge, as it would be of great help in estimating the cause of therapeutic failure [94]. It would allow testing whether treatment failure is indeed due to the emergence of resistant parasites through (i) *in vitro* drug sensitivity using intracellular amastigotes and (ii) molecular characterisation of isolates to compare and to identify the causes of the observed resistance.

### Proposed Actions to Achieve Objective 3

- To encourage epidemiological and clinical groups to provide isolates.
- To encourage clinical groups to support the declaration of cases to official organisations and the access to characterisation of *Leishmania* in immigrant patients, which will provide epidemiological data and help in clinical management and prognosis.
- To share experimental procedures and software tools to enable isolate genomic characterisation and isolate-specific expression differences.
- Characterisation of possible drug resistance mechanisms in relation to cases of treatment failure.
- Training network members in the use of computer tools for genomic characterisation of isolates and analysing changes in gene expression, through training courses and work placements.

## 5. Objective 4: Establishment of a Procedure for the Selection of New Targets and the Development of New Therapeutic Strategies/Alternatives Against Leishmaniasis

Despite the severity of the clinical forms caused by *Leishmania* spp. infections, there are no suitable drugs for their treatment. The available drugs are pentavalent antimonials (Sb^V^), amphotericin B (AmB), deoxycholate, and miltefosine (MIL), all of which have high toxicity and/or require long treatments [95]. Furthermore, all these drugs require parenteral administration, except for MIL. Over the last decade, mainly due to poor adherence to treatment regimens and extensive use in veterinary medicine, resistance to Sb^V^ has increased. MIL is very active against VL and well-tolerated, but its teratogenic potential cannot be ruled out. Paromomycin is currently in clinical trials and is effective, safe, and inexpensive but requires repeated intramuscular injections. Other oral drugs include the azoles and allopurinol, but they have not achieved the required levels of efficacy or safety. Based on available information, single-dose liposomal AmB is currently the best available therapy for VL in India—being more effective and safer than any other treatment, although it appears less effective in Africa—as well as for treating cutaneous and mucosal forms. In addition, its high price and thermal instability continue to restrict its distribution [96]. Liposomal AmB is currently the drug of choice for the treatment of VL in our country, but repeated dosing is required.

Overall, it is important to emphasise the need to develop new drugs with high parasite-selective activity, which means coordinating the efforts of medicinal chemists and parasitologists. For example, chemists should design drugs that are able to interfere with molecules that are unique and essential for the biology of *Leishmania* species (known as virulence factors). This is a rational way to control *Leishmania* by exploiting biochemical differences with the human host. Among potential candidates is the enzyme superoxide dismutase (SOD), which plays a key role in cell biology as a virulence factor and represents the first line of defence against harmful superoxide free radicals. This molecule is unique and specific to trypanosomatids that have an isoform with iron (FeSOD) in its active centre, in contrast to the host SODs cells that contain copper, zinc or manganese. Particular attention is focused on identifying, purifying, and biochemically characterising this bioactive molecule, both at the cellular level and secreted forms in different *Leishmania* species (*L*. *infantum*, *L*. *peruviana*, *L*. *braziliensis* and *L*. *amazonensis*) [97]. This exclusively parasitic enzyme represents an ideal chemotherapeutic target for designing new drugs without adverse events on infected humans or animals [98,99,100]. In addition, their use as antigen fractions in Western blotting and ELISA assays demonstrated their utility as biomarkers in a specific discriminatory diagnosis of different clinical forms of leishmaniasis in both animals and humans [101,102].

Histone deacetylases (HDACs) are involved in the silencing of critical regulatory pathways, including pro-apoptotic programmes, and represent another target of great interest. HDAC inhibitors, mainly those with a hydroxamic acid-based structure, are promising anti-parasitic agents. Recently, two o-alkylhydroxamates with no activity against human HDACs have been reported as a new chemotherapeutic option against *Leishmania*. These molecules demonstrated *in vitro* activity in the micromolar range against intracellular amastigotes and exhibited high efficacy in animal experimental models of VL, CL, as well as naturally acquired CanL, with superior results to pentavalent antimonials, and the possibility of oral administration [103,104]. To continue with the biochemical distinctions between the parasite and its human host, some of its auxotrophies can be exploited. Examples include those related to the amino acid arginine, purines and pyrimidines, and the haem group, among others. Haem is an iron-coordinated porphyrin that serves as a prosthetic group of haemoproteins involved in many fundamental physiological processes. The auxotrophy of *Leishmania* has been attributed to the loss of the synthesis pathway for this essential metabolite in aerobic organisms. Consequently, as with all other trypanosomatid parasites, it must acquire the metabolite from the host organisms [105]. In recent years, there has been significant progress in the field of research identifying proteins associated with porphyrin incorporation and transfer as promising therapeutic targets. *Leishmania* uptake of haem via the plasma membrane transporters LHR1 [106] and LFLVCRb [107] has been well-documented. Both proteins are essential and required for the virulence of this parasite [107,108]. Furthermore, *Leishmania* can obtain the haem, which is bound to haemoglobin present in the host [109]. Following receptor-mediated, clathrin-dependent endocytosis of haemoglobin, this host protein is transported to the parasite lysosome, where release of the haem fraction to the cytosol occurs via the LHR1 transporter [110]. Interestingly, some LHR1 inhibitors that block haem transport kill intracellular *Leishmania* parasites in the nanomolar range [111]. As demonstrated in the study by Campos-Salinas et al. (2011), the ABC transporter LABCG5 also plays a key role in the intracellular transport of haemoglobin-derived haem into the mitochondria [112]. Finally, another ABC transporter, LABCB3, has been identified as a regulator of mitochondrial haem biosynthesis from host precursors. This protein is also required for the biogenesis of cytosolic iron/sulphur complexes and is essential for parasite virulence [113].

Alternatively, natural products and their derivatives have historically been used as therapeutic agents in traditional medicine and are now being identified as sources of antiparasitic agents. Groups such as flavonoids and terpenoids, which are found abundantly in the plant kingdom, are active against a wide range of pathogens, including those of the genus Leishmania. For example, flavonol glycosides from *Delphinium* (*D*.) *gracile DC*, *D*. *staphisagria L*., *Consolida oliveriana*, and *Aconitum napellus* subsp. *lusitanicum* from Tenerife have been shown to be effective against this parasite [114,115]. In the same way, (-)-α-bisabolol, a monocyclic sesquiterpene found in concentrations up to 50% in the essential oil of chamomile [*Matricaria chamomilla* L. (Asteraceae)], has been evaluated *in vivo* by oral and cutaneous routes in laboratory models of naturally acquired canine VL, CL, and CanL, with superior results to pentavalent antimonials because a direct antiparasitic effect associated with a possible immunomodulatory effect [116,117].

In recent years, new therapeutic targets (including *LmjPES*, ID: *LmjF.22.0810*) related to the pathogenicity of the parasite have been identified [118,119]. These findings suggest that they are promising targets for the design of new drugs exhibiting leishmanicidal activity. Also, *LmjPES*, a putative serine/threonine protein kinase of *L*. *major*, is a potential modulator of the Th2-type host immune response and may be relevant for the recovery process of leishmaniasis [118].

### Proposed Actions to Achieve Objective 4

- Compilation of potential targets, strategies of analysis, and therapeutic alternatives generated by the participating groups.
- Design, development, and implementation of a standardised procedure for the assessment of their therapeutic value.
- Include the initiative of other groups working in the development of new therapeutic leishmanicidal drugs in our country.
- Organisation of stays and training courses for network members in gene editing, use of automatic analysis tools, infection models, transcriptome analysis, and the impact of drugs on parasite metabolism.
- Characterisation of the possible mechanisms of resistance to the drugs currently used and the new alternatives.

## 6. Conclusions

Considering the active transmission of *Leishmania* in Spain, it is necessary to articulate a Leishmaniasis Surveillance Network aimed at bringing together the main stakeholders in the research and management of leishmaniasis in our country. Although the present agreement document represents a major step forward in this proposal, it highlights some weaknesses, such as the need for greater operability of the proposed actions, the lack of progress indicators or financial resources, timetable, etc., which must be addressed by the agents involved. The aim of the proposed network must be to mitigate the adverse impact of this vector-borne disease on the health of both humans and animals, following a One Health approach. The establishment of this network will result in several benefits, including the optimisation of diagnosis and clinical care. In addition, it will facilitate the study of risk factors, deepen the knowledge of parasite/vector/vertebrate host relationships, the search of new targets and therapeutic formulas, and consequently the establishment of new prevention and control strategies. The implementation of the One Health approach will promote synergy between the research groups involved, who will benefit from sharing the latest advances in public, veterinary, and environmental health, thereby reinforcing translational research in this vector-borne disease. The advances achieved will finally allow the promotion of a national leishmaniasis control plan from a One Health perspective through the definition of various strategic lines of action. Among these lines, we highlight the organisation of discussion forums and the subsequent drafting of a strategic plan, its objectives, actions, and indicators, with the presentation of this plan to competent Spanish and broader European health surveillance authorities, as well as the WHO.

## Data Availability

No new data were created or analysed in this study. Data sharing is not applicable to this article.
